# Meiotic and developmental competence of growing pig oocytes derived from small antral follicles is enhanced in culture medium containing FGF2, LIF, and IGF1 (FLI medium)

**DOI:** 10.1186/s13048-024-01360-0

**Published:** 2024-03-02

**Authors:** Alexandra Rosenbaum Bartková, Lucie Němcová, Veronika Kinterová, Dominika Radová, František Strejček, Tereza Toralová, Jozef Laurinčík, Radek Procházka

**Affiliations:** 1 Laboratory of Developmental Biology, Institute of Animal Physiology, Genetics of the Czech Academy of Sciences, Liběchov, Czech Republic; 2 Faculty of Natural Sciences and Informatics, Constantine the Philosopher University in Nitra, Nitra, Slovak Republic; 3 Central laboratory Košice, Medirex a.s, Košice, Slovakia

**Keywords:** Follicle size, Oocyte maturation, Developmental potential, Chromatin configuration, Growth factors, MAPK activation, Gene expression

## Abstract

**Background:**

Oocytes of large animal species isolated from small ovarian follicles (< 2 mm) are less competent to support early embryonic development after in vitro maturation and fertilization than their counterparts isolated from medium-sized and preovulatory follicles. This study aimed to assess the effect of a new maturation medium containing FGF2, LIF, and IGF1 (FLI medium) on the meiotic and developmental competence of pig cumulus-oocytes complexes (COCs) derived from the small and medium-sized follicles.

**Methods:**

The growing oocytes were isolated from 1 to 2 (small follicle; SF) and the fully-grown ones from 3 to 6 (large follicle; LF) mm follicles and matured in a control M199 medium with gonadotropins and EGF and the FLI medium enriched by the triplet of growth factors. The matured oocytes were parthenogenetically activated and cultured to the blastocyst stage. Chromatin configuration before and during the culture and MAP kinase activity were assessed in the oocytes. Finally, the expression of cumulus cell genes previously identified as markers of oocyte quality was assessed.

**Results:**

The maturation and blastocyst rates of oocytes gained from LF were significantly higher than that from SF in the control medium. In contrast, similar proportions of oocytes from LF and SF completed meiosis and developed to blastocysts when cultured in FLI. Most of the oocytes freshly isolated from SF possessed germinal vesicles with fine filaments of chromatin (GV_0_) or chromatin surrounding the nucleolus (GV_I_; 30%); the oocytes from LF were mainly in GV_I_ (or GV_II_) exhibiting a few small lumps of chromatin beneath the nuclear membrane. When cultured in the FLI medium for 16 h, an acceleration of the course of maturation in oocytes both from SF and LF compared to the control medium was observed and a remarkable synchrony in the course of chromatin remodeling was noticed in oocytes from SF and LF.

**Conclusions:**

This work demonstrates that the enrichment of culture medium by FGF2, LIF, and IGF1 can enhance the meiotic and developmental competence of not only fully-grown, but also growing pig oocytes and significantly thus expanding the number of oocytes available for various assisted reproductive technology applications.

**Supplementary Information:**

The online version contains supplementary material available at 10.1186/s13048-024-01360-0.

## Introduction

In vitro culture of cumulus-oocyte complexes (COCs) obtained from growing follicles has proven to be a significant technique for studying the processes that regulate the final maturation of mammalian oocytes. In various species, including humans, matured and fertilized oocytes cultured in vitro have successfully developed into healthy progenies. However, it is still observed that the developmental potential of in vitro matured oocytes is lower compared to those matured naturally within the body. Approximately 30–40% of in vitro matured oocytes in large animals can reach the blastocyst stage capable of supporting embryonic development [1, 2]. In recent times, there have been reports from multiple groups about innovative modifications made to in vitro maturation (IVM) systems. These modifications involve manipulating the intracellular cyclic adenosine monophosphate (cAMP) concentration or adding hormone and growth factor supplements. It has been observed that these changes greatly enhance the developmental competence of cultured oocytes from mice, cattle, and pigs [3–6].

Oocyte developmental competence refers to the ability of a fully grown oocyte to be fertilized, develop into an early preimplantation embryo, and support development until birth [7]. The acquisition of complete oocyte developmental competence is a gradual process that occurs in stages alongside ovarian follicle growth during oogenesis [8, 9]. Initially, the oocytes gain the capacity to reach metaphase II and undergo maturation. In the later stages of follicular development, the oocytes gain the ability to support early embryo development. As folliculogenesis progresses toward ovulation, the oocytes acquire full developmental competence, enabling them to support development until birth. The final phase of oogenesis is referred to as “oocyte capacitation” and involves structural changes in organelles within the oocyte itself, along with various molecular events. These changes not only occur within the oocyte but also affect the surrounding cumulus cells, allowing for a response to luteinizing hormone/ receptor (LH/LHR) signaling during ovulation [10].

In large animal species and humans, the oocytes progressively mature in smaller or medium-sized antral follicles until they are fully developed a few days before ovulation [11]. Consequently, the follicle’s diameter in adult cycling females and even prepubertal animals serves as a reliable measure of oocyte readiness to complete their maturation process and facilitate embryonic growth. Numerous studies in various animal species, including cows [9, 12], sheep [13], goats [14], horses [15], monkeys [16] and humans [17, 18] have reported a strong association between follicle size and the meiotic and developmental competence of oocytes. In relation to our model organism, the pig, a study by Töpfer et al. (2016) has also confirmed that oocytes isolated from follicles larger than four millimeters exhibit higher developmental competence [19].

The achievement of oocyte developmental competence is dependent on several factors. The key molecular factors are the activation of the maturation-promoting factor (MPF) in the oocyte cytoplasm and the transition from the G2 phase to the M-phase of the cell cycle, both of which are associated with the resumption of oocyte meiosis [20]. When investigating why meiotic failure occurs in growing porcine oocytes, it was found that small oocytes have three major problems compared to their larger counterparts: an inactive mitogen-activated protein kinase (MAPK) cascade, a lack of cyclin B synthesis, and insufficient levels of cdc2 kinase [21, 22]. A study performed in pig oocytes found that inadequate presence of Cdc2 was the primary cause of meiotic failure [21]. By introducing mRNA encoding c-Mos, which activates the MAPK cascade, cyclin B2 and Cdc2 into the oocyte, it was shown that overexpression of Cdc2 while suppressing the Wee1B kinase - responsible for MPF inactivation - resulted in smaller porcine oocytes (95–105 μm 0.4–1 mm follicle) achieving a GVBD rate of 70%, similar to full-size oocytes [21].

The achievement of developmental competence is also influenced by cumulus cells because they have a key role in supporting and communicating with the oocyte [23]. They are necessary for the development and maturation of the oocytes. They provide metabolic assistance to the oocyte by facilitating communication through gap junctions, ensuring an ample supply of ATP [24]. It was shown that cumulus cells play a key role in maintaining adequate levels of ATP in the oocyte, which is essential for meiotic progression and developmental competence. In addition, the involvement of cumulus cells in regulating oocyte maturation is crucial. They generate different growth factors, including epidermal growth factor (EGF) and transforming growth factor-alpha (TGF-α), that stimulate oocyte maturation. By binding to receptors on the surface of the oocyte, these growth factors initiate internal signalling pathways that facilitate meiotic resumption and cytoplasmic maturation [25]. The smooth progression of these processes is ensured by the presence of cumulus cells, which also regulate them through the transcriptomic activity of genes and miRNAs that are expressed differently [26, 27].

A significant increase in maturation and developmental competence has been recently attained in fully gown pig oocytes cultured under in vitro conditions by supplementation of medium containing EGF, gonadotropins (FSH, LH) and three additional cytokines, leukemia inhibitory factor (LIF), insulin-like growth factor-1 (IGF-1), and fibroblast growth factor-2 (FGF-2) [6, 28]. By using this enriched medium known as FLI medium, it becomes feasible to substitute undefined protein sources like fetal calf serum or follicular fluid with polyvinyl alcohol or bovine serum albumin without decreasing maturation competence [29].

The goal of this research was to investigate if using FLI medium could enhance the maturity and developmental capability of immature growing pig oocytes obtained from small antral follicles. Various factors such as chromatin remodeling, activation of MAPK3/1, resumption and completion of meiosis, and ability to develop into blastocysts after parthenogenetic activation were evaluated in both growing and fully grown oocytes cultured in control and FLI medium.

## Materials and methods

All the procedures with animals were performed according to good veterinary practice for animal welfare according to the relevant Czech laws (No. 246/1992 and 419/2012). All materials were purchased from Thermo Fisher Scientific (Waltham, MA, USA) unless otherwise stated. All chemicals were purchased from Merck (Kenilworth, NJ, USA) unless otherwise stated.

### Collection and culture of cumulus-oocyte complexes

Porcine ovaries of prepubertal gilts, 6–8 months old and 90–120 kg in weight, were collected at a local abattoir as a waste material and transported to the laboratory in a thermo-flask at 38 °C. The contents of small (1–2 mm in diameter) and medium-size antral follicles (3–6 mm in diameter) were aspirated with a syringe connected to a 20 G needle, pooled in a test tube, and allowed to sediment for 10 min. The sediment was washed twice with HEPES buffered porcine X medium (PXM-Hepes) [30], placed in a Petri dish, and the COCs were collected with a pipette. Only COCs surrounded by a compact multi-layered cumulus were selected for experiments. Groups of 50 COCs were cultured for 44 h in four-well dishes (Nunclon, Roskilde, Denmark) in 0.5 ml of culture medium at 38.5 °C in a humidified atmosphere of 5% CO_2_. The diameter of some COCs and denuded oocytes isolated from small and medium follicles was measured by Axiocam 208 camera using Zen 3.6 software (Zeiss).

### Culture media and reagents

The COCs were cultured either in a control M199 or modified FLI medium [5]. The composition of the control and FLI medium is given in Table 1.

**Table 1:** The composition of the control and FLI medium

Component	Supplier	Control medium	FLI medium
TCM199	Sigma, M7528	TCM199	TCM199
Sodium pyruvate	Sigma, P4562	0.2 mM	0.2 mM
L-glutamin	Sigma, G8540	6.85 mM	6.85 mM
Cysteine	Sigma, C7352	0.57 mM	0.57 mM
Gentamycin	Roth, 0233	50 µg/ml	50 µg/ml
BSA	Sigma, A7030	1 mg/ml	1 mg/ml
PMSG	Prospec^1^, HOR-272	10 IU/ml	10 IU/ml
hCG	Prospec^1^, HOR-250	10 IU/ml	10 IU/ml
EGF	PeproTech^2^,AF-100-15	10 ng/ml	10 ng/ml
human LIF	Merck, LIF1005	−	2 µl/ml
human IGF1	PeproTech, AF-100-11	−	20 ng/ml
human FGF2	Sigma, F0291	−	40 ng/ml

^1^Prospec, Rehovot, Israel; ^2^PeproTech, London, England.

### Assessment of oocyte maturation

To assess their nuclear maturation, oocytes after 44 h of cultivation were stripped of cumulus cells by vortexing. The degenerated oocytes (< 5%) were excluded from the evaluation and the remaining ones were mounted on slides and fixed in an acetic acid-ethanol solution (1:3) for 48 h. Oocytes were then stained with 1% orcein and observed with a light microscope. To confirm the achievement of metaphase II, we focused on determining chromosomes in the equatorial plane and excluded the polar body.

### Parthenogenetic activation and culture of embryos

For parthenogenetic activation, cumulus cells were removed from COCs by pipetting and washed twice in PXM-HEPES. Oocytes with recognized polar body were activated by exposure to 10 µM ionomycin in PXM-HEPES for 5 min. After that, they were washed twice in porcine zygote medium 3 (PZM 3) [31] supplemented with 2 mM 6-DMAP and cultured for 5 h at 38.5 ˚C under a 5% CO_2_ atmosphere. Around 50 putative parthenotes were washed twice in PZM 3 and cultured for 6 days in 4-well dishes in 1 ml of PZM 3 medium at 38.5˚C under a 5% CO_2_ atmosphere. After 40 h, the cleavage of embryos was assessed and after 144 h, the ability of embryos to reach the blastocyst stage was analyzed.

### Immunochemistry

The denuded oocytes collected right after isolation from follicles or after 16, 24, 44 h of maturation were fixed in 4% paraformaldehyde for 40 min at room temperature. Fixed oocytes were processed immediately or stored in PBS supplemented with 2% BSA (PBS/BSA) at 4 °C. After washing in PBS/BSA, oocytes were permeabilized and blocked with PBS supplemented with 0.5% TritonX-100 and 2% BSA for 90 min at room temperature. All subsequent steps were done in PBS/BSA. Oocytes were incubated with mouse anti-lamin antibody (Sigma-Aldrich SAB4200236, St. Louis, MO), diluted 1:100 in PBS/BSA, overnight at 4 °C. After washing twice in PBS/BSA for 30 min at room temperature, oocytes were incubated with secondary goat anti-mouse antibody conjugated with Alexa Fluor 594, diluted 1:350 (Invitrogen, Eugene, OR) in PBS/BSA for 2 h at room temperature in the dark. After washing, the DNA of the nuclei was stained and the oocytes were mounted in Vectashield Vibrance Antifade Mounting Medium with DAPI (4’, 6-Diamidine-2’-phenylindole dihydrochloride, Vector Laboratories, Peterborough, UK). Controls of immunostaining specificity were carried out by omitting the primary antibody. The samples were examined using Leica TCS SP5 (Leica Microsystems AG, Wetzlar, Germany) and the images were processed using Image J software (National Institute of Mental Health, Bethesda, MD, USA).

### Immunoblotting

At the selected culture time (GV – 0 h, MI – 28 h, MII – 42 h) groups of 50 denuded oocytes were washed in PBS and solubilized in Laemmli buffer containing 2% sodium dodecyl sulfate (SDS) and 5% 2-mercaptoethanol. Samples were boiled at 100 °C for 3 min and stored frozen at − 20 °C. Subsequently, proteins were separated in 10% acrylamide/SDS gels and transferred to Immobilon-P membranes (Millipore, Bedford, MA, USA). Membranes were blocked in 5% low-fat dry milk in Tris-buffered saline (TBS) with 0.5% Tween 20 for 2 h at room temperature, and then incubated with a primary antibody diluted 1:1000 in 2.5% BSA in TBS-Tween, at 4 °C overnight. The primary antibodies were p-ERK and ERK1 (detecting MAPK3/1), both from Santa Cruz Biotechnology (Santa Cruz, CA, USA). The secondary antibodies (Amersham ECL anti-mouse or anti-rabbit IgG, GE Healthcare, Little Chalfont, UK) were diluted 1:5000 in 2.5% BSA in TBS-Tween. The membranes were incubated with the secondary antibody for 1 h at room temperature and then washed intensively in TBS-Tween. The immune reaction was detected by enhanced chemiluminescence (Pierce, Rockford, IL, USA) according to the manufacturer’s instructions. The intensity of the specific bands on the blots was analyzed by scanning densitometry using the free software Image J Version 1.29 (National Institute of Mental Health, Bethesda MD, USA).

### Expression analysis of cumulus cells genes related to oocyte quality RT-qPCR

For our experiments, we utilized the cumulus cells of mature oocytes from individual experimental groups. We collected cumulus cells from 20 oocytes, washed them three times in PBS, and then froze them at -8​0 °C ​using buffer from the Ambion RNAqueous-Micro Kit (Thermo Fisher Scientific, Waltham, MA, USA). A group of predicted target genes related to oocyte development and cumulus cells expansion was selected and analyzed in the different experimental groups. Primers for the selected target and quality-related genes were designed using the software Beacon Designer v. 8.21 and listed in Table 2. The one-step RT-qPCR was conducted in a RotorGene 3000 cycler (Corbett Research, Mortlake, Austria) using the QIAGEN OneStep RT-PCR Kit (Qiagen, Germany) in a 20 µl reaction mixture containing 4 µl 5 X reaction buffer, 0.8 µl dNTP mix (10 nM stock), 0.4 µl forward and reverse primers (20 nM stock), 0.125 µl RNasin (20 IU/ml stock, Promega), 0.8 µl enzyme mix, 0.8 µl EvaGreen (Biotium, CA, USA), 3 µl RNA, and nuclease-free water. The reaction conditions were as follows: reverse transcription at 50 °C for 30 min., initial denaturation at 95 °C for 15 min, followed by PCR cycles consisting of denaturation at 94 °C for 15 s, annealing at a temperature specific for each set of primers (Table 2) for 15 s and extension at 72 °C for 20 s; and a final extension at 72 °C for 5 min. Fluorescence data were acquired at the end of each extension step. Products were verified by melting analysis and gel electrophoresis on 1.5% agarose gel with MidoriGreen Direct (Nippon Genetics, Dueren, Germany). Comparative analysis software (Corbett Research) was used for gene expression analyses after normalization to the geometric mean of *YWHAG* and *TBP* as internal control genes for cumulus cells.

**Table 2:** List of primers used for real-time RT-PCR

Gene trancript	Primers 5‘ –– 3‘	Amplicon length (bp)	T_an_ (°C)	GenBank accession number
*AKT1*	TAC TCC TTC CAG ACC CAC GA CGG AGT GCA GGT AGT CCA AG	157	53	NM_001159776.1
*ATP6*	AAT TCC TAT GCT CGT AAT ATG TTG AGT AGT GCT AAT	141	27	NC_000845
*CCND2*	CAG TGC TCC TAC TTC AAG ACC TCT TCT TCA CAC TTC	116	58	NM_214088.1
*HAS2*	GAA GTC ATG GGC AGG GAC AAT TC TGG CAG GCC CTT TCT ATG TTA	407	54	NM_214053.1
*PCNA*	TAA TGC AGA CAC CTT GGC ACT GCA AAT TCA CCA GAA GGC ATC	153	55	NM_001291925.1
*TBP*	ATA GCC TTC CAC CTT ACG CTC ATA GGC TGT GGA GTC AGT CCT	115	58	XM_021085483.1
*YWHAG*	CAG CCC ACT CAC CCC ATT AG TGC TGA TCG CTT GTC CAG AG	218	58	XM_005661962.3

T_an_ - annealing temperature

### Statistical analysis

Each experiment was performed in at least three replicates. Oocyte and COC diameter, meiotic competence and the development of embryos, and intensity of the specific bands on immunoblots were analyzed by one-way ANOVA with Holm-Sidak’s post-test (SigmaPlot 12.0, London, UK). The differences in chromatin remodeling between SO and LO were compared by student t-test. Error bars indicate the standard error of the mean (SEM). Probability values < 0.05 were considered to be statistically significant.

## Results

### Oocyte and COC diameter

The diameter of oocytes derived from the large follicles (LF; 3-6 mm) was 122.4 ± 0.5 μm and was significantly different from the diameter of oocytes derived from the small follicles (SF, 1-2 mm) (113.4 ± 0.4 μm; *P* < 0.01). The cumulus cell compartment surrounding the oocytes from LF was also significantly larger than that of from SF since the diameters of the COCs were 350 ± 0.5 vs. 310 ± 0.35 μm, respectively (*P* < 0.05). The diameter of matured oocytes from LF was 151.2 ± 0.6 μm in FLI and 141.9 ± 0.95 μm in control media. The diameter of matured oocytes derived from the SF group was 139.6 ± 2.1 μm in FLI and 128.1 ± 1.4 μm in control media.

### Maturation of oocytes in control and FLI medium

**Fig. 1:**
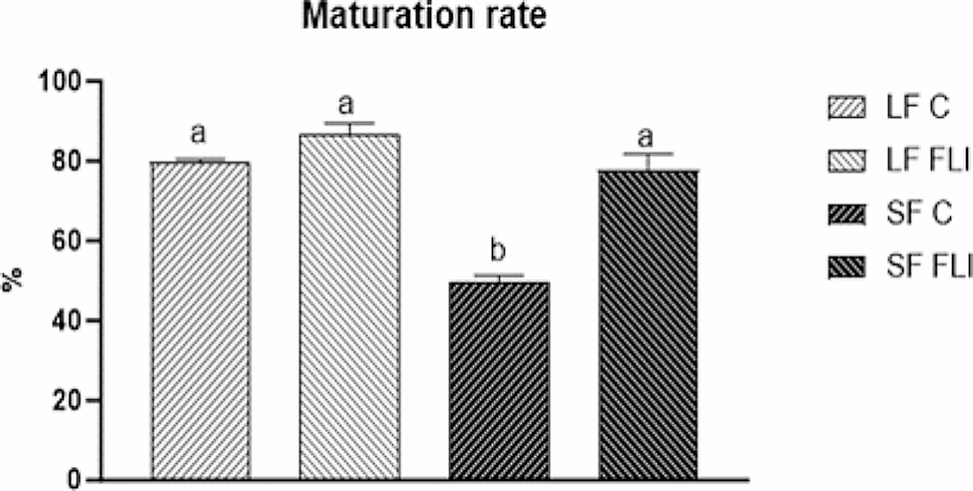
Effect of FGF2, LIF and IGF1 (FLI medium) on the maturation of pig oocytes derived from small and large follicles. Different superscripts above the column indicate significant differences (*P* < 0.05)

Oocytes were scored for GV, GVBD (mostly comprising oocytes at the metaphase I stage and few oocytes at late diakinesis, anaphase I or telophase I) and for metaphase II stage (MII). The maturation rate of oocytes from LF to MII stage was significantly higher than that of oocytes from SF (79.7 ± 0.47 vs. 49.6 ± 1.6%; *P* < 0.01) in control medium. In contrast, similar proportions of oocytes from LF and SF completed meiosis when cultured in FLI medium (86.9 ± 2.4 vs. 77.7 ± 4.1%; *P* > 0.05) (Fig. 1) (Supplemental Table 1).

### Developmental potential of oocytes cultured in control and FLI medium

**Fig. 2:**
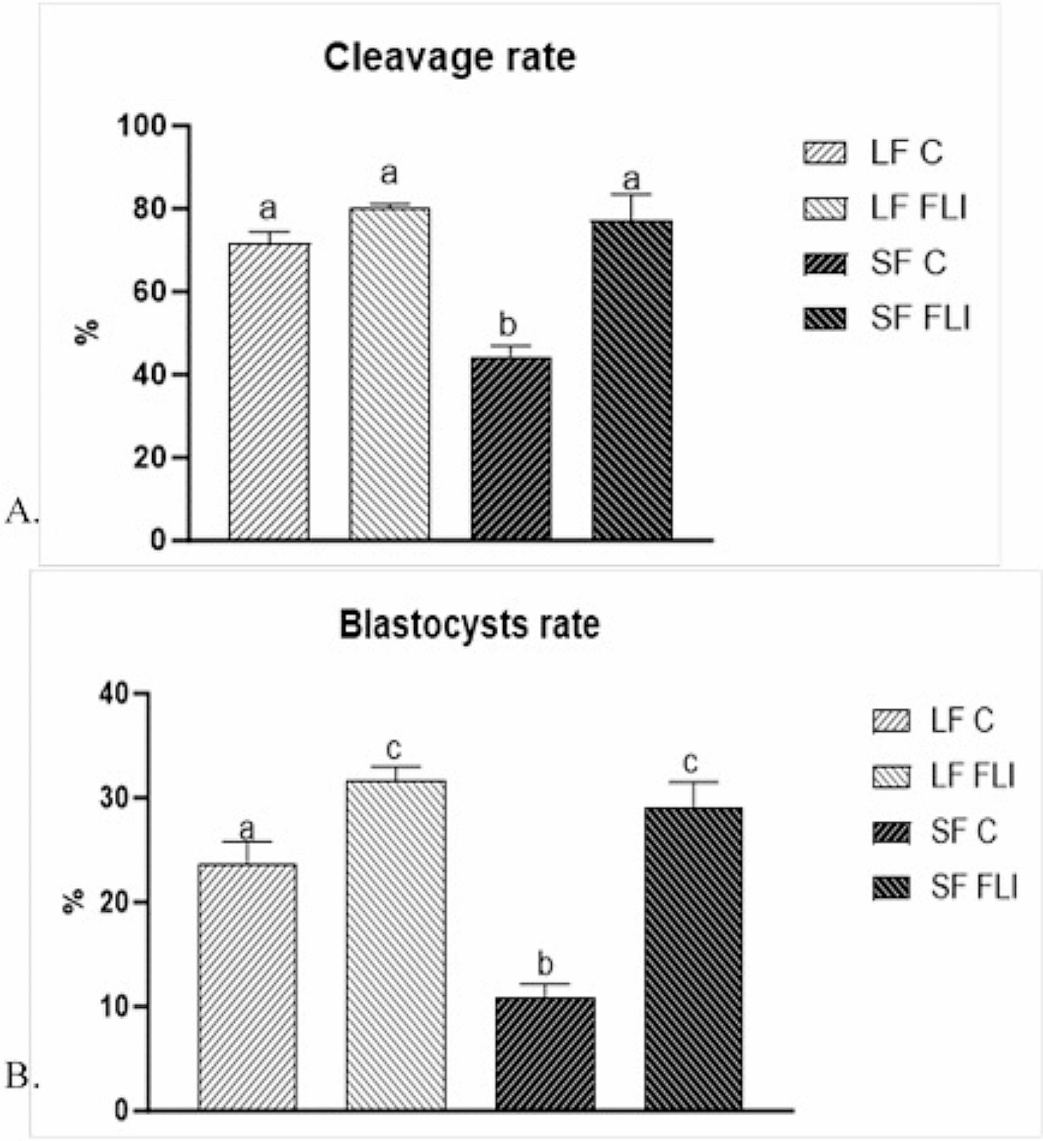
Effect of FLI medium on the developmental potential of porcine parthenogenetic embryos. (**A**) Cleavage rates and (**B**) Blastocyst rates of embryos derived from oocytes isolated from small and large follicles. Different superscripts above the column indicate significant differences (*P* < 0.05)

The cleavage rate was significantly higher in oocytes from LF than from SF (71.6 ± 2.6 vs. 44.4 ± 0.84%; *P* < 0.05) when COCs were cultured in the control medium whereas it was similar for both oocyte categories (80.0 ± 3.2 and 77.3 ± 8.2% for LF and SF, respectively; *P* < 0.05) when cultured in FLI medium (Fig. 2A). Consequently, the blastocyst rates were higher in LF than in SF (23.6 ± 1.6 and 10.9 ± 1.9%, respectively; *P* < 0.05) when oocytes were cultured in the control medium whereas they were equal when oocytes were cultured in the FLI medium (31.6 ± 1.36 and 29.2 ± 2.0%, respectively; *P* < 0.05) (Fig. 2B) (Supplemental Table 2).

### Dynamic changes in chromatin configuration during culture of oocytes in control and FLI medium

**Fig. 3:**
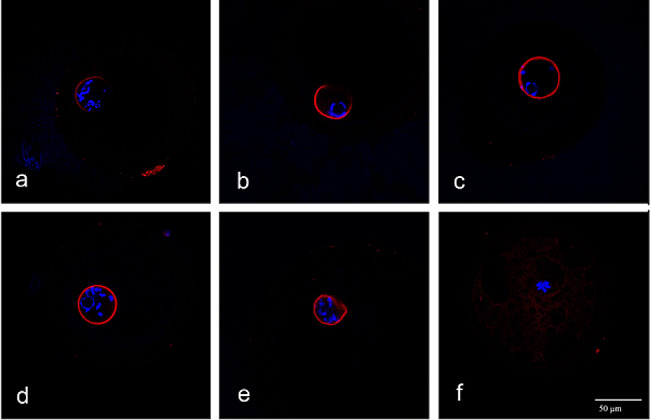
Examples of chromatin configurations in SO and LO before and during the culture (16 h) in vitro. DNA(DAPI)-blue; Lamin-red. (**a**) GV_0_; (**b**) GV_I_; (**c**) GV_II_; (**d**) GV_III_; (**e**) GV_IV_; (**f**) GVBD.

**Fig. 4:**
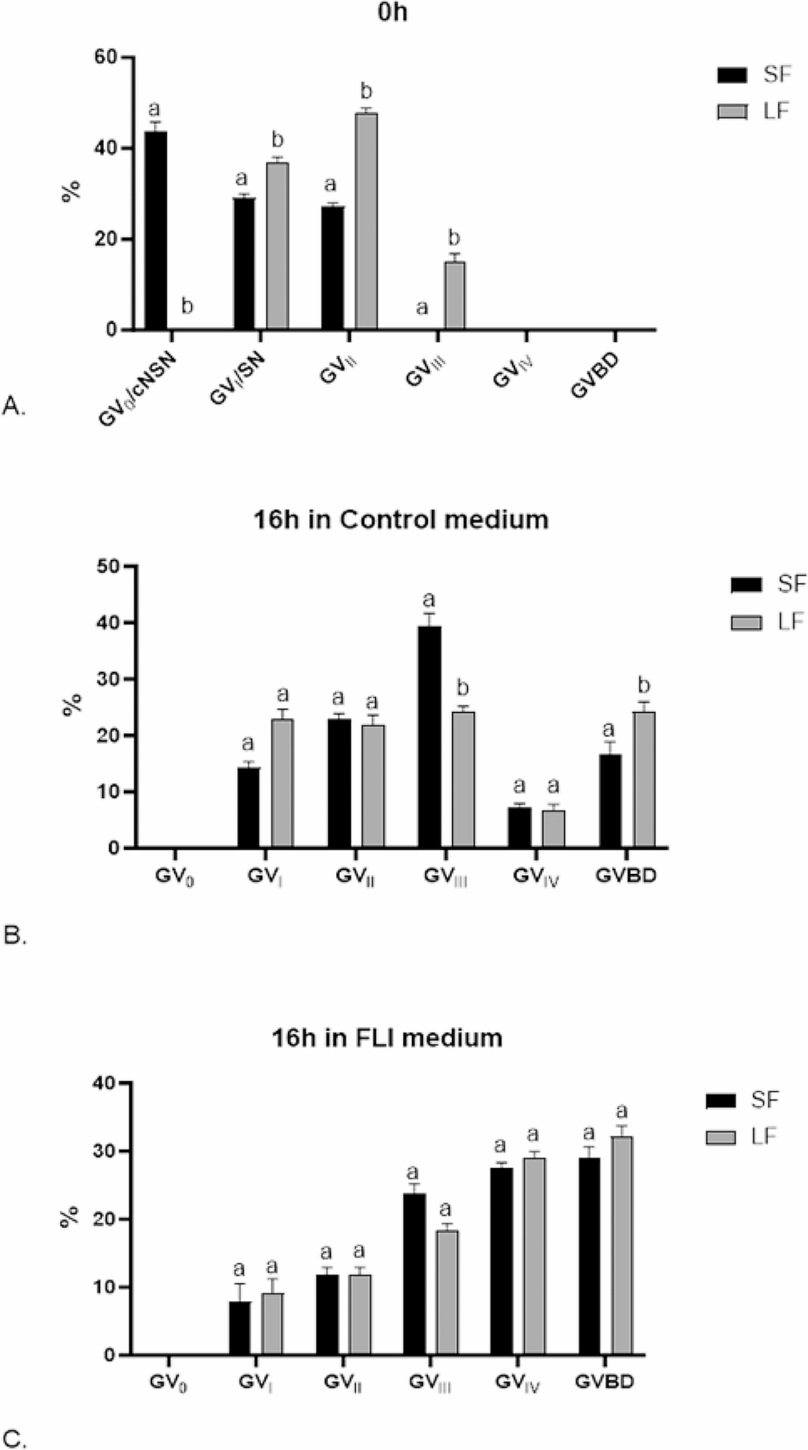
Effect of FLI medium on chromatin configuration of oocytes derived from small and large follicles and cultured for 16 h. GV: germinal vesicle; GVBD: germinal vesicle breakdown. Different superscripts above the column indicate significant differences between oocytes derived from small and large follicles

Most of the oocytes from SF immediately after isolation displayed the non-surrounding pattern of chromatin in the nucleus, showing fine filaments of chromatin within the nucleoplasm (GV_0_; 43.8%; Fig. 3a), some oocytes had undergone the transition from the non-surrounding to the nucleolus-surrounding pattern of chromatin organization and displayed a dense rim of condensed chromatin around nucleolus (GV_I_; 39%; Fig. 3b) that was sometimes supplemented by a few lumps of chromatin beneath the nuclear membrane (GV_II_; 27.2%; Fig. 3c). As expected, virtually all of the oocytes from LF had undergone the transition from the non-surrounding to the nucleolus-surrounding pattern of chromatin organization and displayed GV_I_ (36.9%), GV_II_ (47.9%) or GV_III_ (a less distinct rim of chromatin around nucleolus supplemented by filaments of condensed chromatin in the nucleoplasm; 15.2%; Figs. 3d and 4A).

When cultured in the control medium for 16 h, virtually all oocytes from SF left the GV_0_ stage and displayed more condensed patterns of chromatin, the GV_III_ being the most frequent stage (39.3%). A small portion of the oocytes from SF underwent GVBD (16.5%; Fig. 3f) during this period of culture. The progress of maturation in the control medium was relatively slow in oocytes from LF, most oocytes remained still in the GV_I_ -GV_III_ stage. Nevertheless, there were significant differences between SF and LF in the two chromatin stage categories: more oocytes from LF underwent GVBD (24.3 vs. 16.5%; *P* < 0.05) and fewer oocytes from LF remained in the GV_III_ stage (24.2 vs. 39.3%; *P* < 0.05) (Fig. 4B).

When cultured in the FLI medium for 16 h, two well-marked features emerged from chromatin shape assessment: (1) an acceleration of the course of maturation in both SF and LF compared to the control medium. Most of the oocytes of both size categories proceeded to GV_III_ (SF 23.7%; LF 18.4%) or GV_IV_ (SF 27.6%; LF 28.9%) or even underwent GVBD (SF 28.9%; LF 32.1%), which represents a significant shift to more advanced stages of maturation. (2) remarkable synchrony in the course of maturation between SF and LF. There were no significant changes between SF and LF in the proportions of oocytes in the specific stages of maturation (GV_I_-GVBD) (Fig. 4C). This indicates that the oocytes from SF not only matured faster in FLI than in the control medium, but they surprisingly made up for the delay in maturation displayed at the beginning of the culture.

### Activation of MAPK3/1 in SF in control and FLI medium

**Fig. 5:**
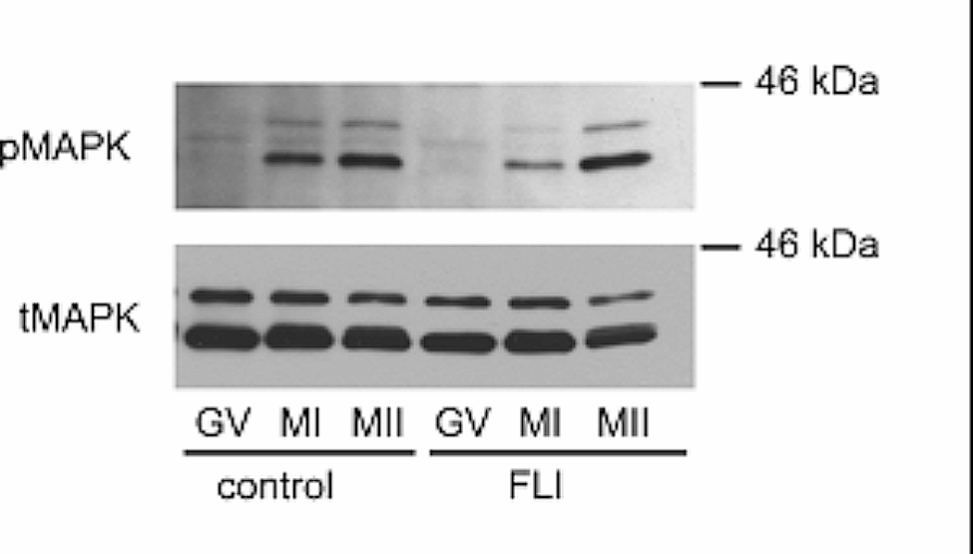
Activation of MAPK3/1 in oocytes derived from small follicles and cultured in control and FLI medium. The experiment was repeated three times with the same results. A representative immunoblot. GV: germinal vesicle; MI: metaphase I; MII: metaphase II.

We tested a hypothesis that lower rates of maturation of SF in the control medium are caused by an inability to activate oocyte MAPK3/1 and downstream pathways necessary for the resumption of meiosis. For this reason, we assessed the activity of MAPK3/1 in SF cultured in control and FLI medium at the maturation stages known to exhibit the high activity of this kinase, i.e. in MI and MII stages. There was a negligible signal of phosphorylated kinase at the GV stage, but in both types of media, the kinase became activated in the MI and MII stages. Therefore, in the oocytes isolated from follicles of 1.5–2 mm in diameter, the lower rates of maturation were not caused by a failure in activating MAPK3/1 (phosphorylated MAPK3/1 to total MAPK3/1 intensity ratio: GV – 1.15 ± 0.20; control medium MI – 4.83 ± 0.54; control medium MII – 10.14 ± 2.67; FLI medium MI – 3.76 ± 0.45; FLI medium MII – 14.17 ± 0.97, Fig. 5).

### mRNA expression analysis of CCs cultured in control and FLI medium

During this analysis, we focused on examining the gene expression of cumulus cells in mature oocytes.

**Fig. 6:**
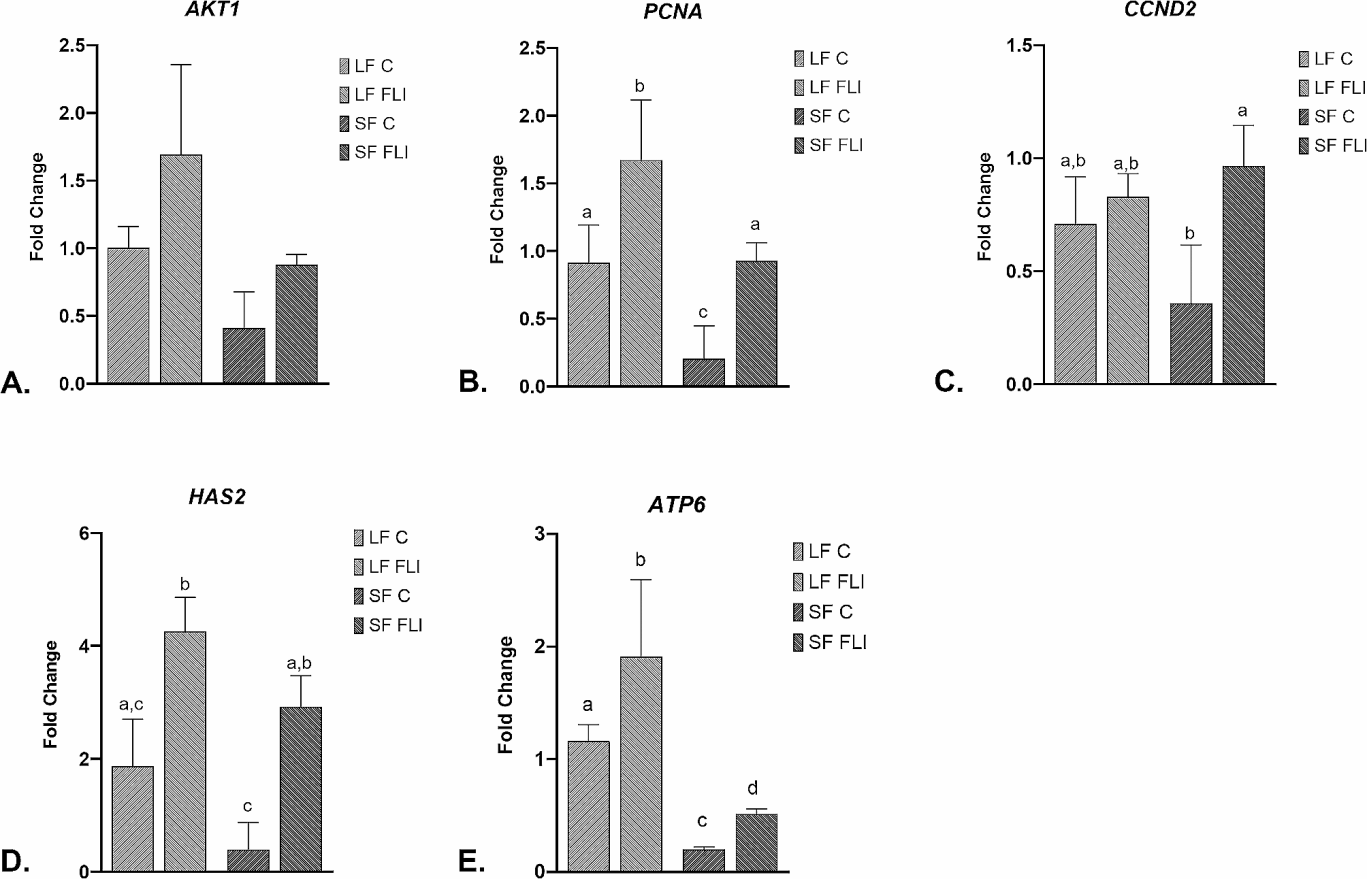
Gene expression in cumulus cells (CCs) cultured in control and FLI medium. *AKT1*(**A**), *PCNA*(**B**), *CCND2*(**C**), *HAS2*(**D**), *ATP6*(**E**). Different superscripts above the column indicate significant differences (*P* < 0.05)

The expression of certain genes in the SF FLI group showed a noticeable increase compared to the SF control group. Additionally, gene expression in SF FLI approached that of the LF control group. The expression of serine/threonine protein kinase *AKT1* was higher in FLI groups, but without significant differences (Fig. 6A). The transcript levels of mitochondrially encoded ATP synthase membrane subunit 6 (*ATP6)* signaling pathway genes were significantly higher in CCs cultured in FLI medium in comparison to corresponding oocyte group cultured in control media (Fig. 6E). In CCs derived from oocytes matured in FLI medium, there was a significant increase in mRNA expression of the Hyaluronan synthase 2 (*HAS2)* gene (Fig. 6D). Furthermore, transcripts related to proliferation (*PCNA*) were also significantly upregulated in the presence of FLI medium, and expression of Cyclin D2 (*CCND2)* was significantly lower in the SF C group (Fig. 6B, C, resp.).

## Discussion

Most of the oocytes that can be collected from the ovaries of large animals in slaughterhouses by aspiration or dissection are located in small follicles. These oocytes are still growing and have chromatin in a diffuse or partially condensed state, resulting in limited abilities for meiosis and development. Various approaches have been proposed to address this problem and increase oocyte growth potential specifically in preovulatory oocytes cultured in vitro. One approach to improve or maintain communication between cumulus and granulosa cells with the oocyte is to modify the function of gap junctions. By adding cAMP to the culture medium, gap junctional communication between oocytes and cumulus cells can be prolonged, leading to improved meiotic competence in bovine and porcine oocyte cultures [32, 33]. The addition of factors that are produced in growing ovarian follicles by oocytes or granulosa cells to the culture medium holds promise as a relatively simple method to increase oocyte competence. For example, the combination of amphiregulin (AREG) with cAMP, bone morphogenetic protein 15 (BMP15) and growth differentiation factor 9 (GDF9) resulted in improved oocyte maturation and blastocyst formation in low-competent porcine oocytes obtained from small follicles [34]. Similarly, the developmental competence of ovine oocytes was increased when C-type natriuretic peptide was combined with AREG and prostaglandin E2 [13]. After analyzing this information, we hypothesized that the presence of additional growth factors during follicular development, which has been shown to increase the efficiency of fully mature oocytes, may also support the meiotic and developmental capabilities of developing oocytes in in vitro culture. Findings from our research indicate that introducing a combination of FGF2, IGF1, and LIF into the culture medium significantly increases the meiotic and developmental potential of growing porcine oocytes, rivaling fully-grown oocytes.

To our knowledge, this is the initial report suggesting that this combination of growth factors can improve the developmental capacity of growing mammalian oocytes during the in vitro maturation period. Studies to date have shown that FGF2, IGF1, LIF and similar growth factors increase the maturity and developmental potential of fully developed mammalian oocytes. Specifically, it has been well established that epidermal growth factor or EGF-like factors promote both maturation and developmental competence in various animal species, including pigs, cattle, sheep, horses, cats, dogs, rodents, monkeys, as well as human oocytes [4]. Barros et al. found that the addition of FGF2 to the culture medium resulted in improved nuclear maturation, higher cumulus cell survival and increased extracellular matrix quality in bovine COCs [35]. The addition of IGF1 has been shown to increase oocyte maturation, leading to improved embryo development and blastocyst formation [36–38]. LIF supplementation in the medium was also found to increase the nuclear maturation of porcine and bovine oocytes as well as blastocyst development [39–41]. The use of FLI medium, consisting of FGF2, LIF and IGF1, allows the elimination of unspecified protein sources such as fetal calf serum or follicular fluid. Instead, they can be replaced with polyvinyl alcohol or bovine serum albumin (BSA), all while maintaining the ability to ripen. Interestingly, this modification in the culture medium significantly increases the quality of porcine oocytes to a level comparable to those that mature naturally in vivo [6, 28, 42]. We show that this relatively simple approach to promote oocyte competence may increase the efficacy of various biotechnological and medical applications, especially in species such as large animals or humans, where the number of fully-grown oocytes available is limited for physiological, medical or ethical reasons.

The mechanisms of growth-factor enhanced oocyte competence have not been completely elucidated up to now. It appears that an important role in the growth factor/cytokine-promoted oocyte competence play protein kinases such as MAPK3/1 and AKT which regulate proliferation, survival, and gene expression in oocyte surrounding cumulus cells, the function of gap junction between oocyte and cumulus cells as well as translation of specific proteins in oocytes [5, 43, 44]. All these functions are closely related to the establishment of oocyte developmental competence.

Various changes in oocyte morphology and function are associated with the acquisition of meiotic competence at the final stage of oocyte growth. These changes include various aspects such as cytoplasmic organelles, nuclear membrane, cytoskeletal organization, gap junction communication, metabolism, chromatin remodeling, nuclear compaction and silencing of transcriptional activity at a global level [45–47]. The maturation and readiness of oocytes in all mammalian species are associated with the transformation of their chromatin pattern. This involves a shift from a dispersed nucleolus to a compact nucleolus that is surrounded by chromatin. Oocytes that are ready for final maturation, fertilization, and early embryonic cleavage are characterized by condensed chromatin and cessation of transcriptional activity. At a specific developmental stage unique to each species, transcriptional activity resumes. In vivo, oocytes can remain in this state for several days until they are selected for final maturation or undergo atresia along with the destruction of the entire follicle [11]. Several studies confirmed this correlation [48–50]. According to several studies, it has been suggested that the transcriptional activity in the nucleus of oocytes gradually decreases as chromatin condenses, eventually stopping completely when oocytes reach full maturity [47, 51, 52]. However, recent techniques such as RNA-seq have revealed that fully grown oocytes exhibit transcriptional activity during meiotic maturation [49, 53, 54].

In pigs, the majority of oocytes extracted from 1 to 2 mm follicles showed an NSN (non-surrounded nucleolus) configuration for their chromatin (approximately 50%). Approximately 30% of these oocytes had partially condensed chromatin, while 10% were categorized as SN (surrounded nucleolus). The remaining oocytes were considered defective due to premature chromatin condensation (GV_0_). Based on a study conducted by Pan et al. in TCM199 medium containing FSH, LH, and EGF, only half of these oocytes were able to successfully mature to the MII stage-consistent with our findings. However, our research shows that by using FLI medium growth factors/cytokines, we can reverse the abnormal chromatin condensation in growing oocytes and facilitate a smooth transition to the MI stage.

Small oocytes obtained from porcine small follicles were found to lack the ability to activate cdc2 and MAPK, as well as to phosphorylate ribosomal S6 kinase (RSK), a downstream target of MAPK. Consequently, these oocytes were unable to resume meiosis when cultured in vitro [21, 22]. Thus, we hypothesized that the limited rate of maturation observed in small oocytes cultured with the control medium compared to FLI could be attributed to the absence of MAPK activation. However, the findings of our experiment clearly showed that this assumption was incorrect, as small oocytes activated MAPK regardless of the type of medium used. The reason for this discrepancy with previous data is probably the different origin of the considered small oocytes. In previous research, small non-viable oocytes were separated from follicles between 0.5 and 1.5 mm [22] or 0.4 and 1 mm in size [21]. However, in our study we extracted small oocytes from follicles measuring 1 to 2 mm, resulting in oocytes with a significantly larger diameter of approximately 113 μm compared to the range of 95–105 μm found previously [21].

Oocyte development and maturation are influenced by the important role played by the cumulus cells. These specific cells encapsulate the oocyte and offer crucial support and nourishment during the folliculogenesis. Gene expression in cumulus cells of oocytes matured using FLI medium was significantly higher compared to those matured using the control medium. A substantial increase in gene expression was observed in a group of oocytes obtained from small follicles in comparison to large follicles that underwent maturation in FLI medium, indicating an increase in their developmental competence. The role of AKT1 expression in FLI groups is crucial for several important cellular processes such as proliferation, metabolism, cell differentiation and survival [55].

Research has revealed that activation of AKT1 promotes the expansion of cumulus cells by controlling the expression of hyaluronan synthase 2 (HAS2), an enzyme involved in the synthesis of hyaluronic acid. HAS2 overexpression and cumulus expansion lead to improved oocyte quality and increased fertilization rate [25]. In addition, overexpression of HAS2 in cumulus cells of human oocytes led to better quality embryos [56, 57].

The AKT1 has also been found to regulate the expression of proliferating cell nuclear antigen, a marker indicating cumulus cell proliferation [58]. The active proliferative state of cumulus cells, crucial for oocyte development and support, is demonstrated by increased expression of PCNA. The upregulation of PCNA may be influenced to some extent by the activation of AKT1, as previously explained. Interestingly, recent research has shown that CCND2 expression can be found in the cumulus cells of fully developed oocytes [59]. This discovery suggests a potential involvement of CCND2 in the control of cumulus cell progression and growth during the cell cycle. Further studies are needed to better understand the exact roles of CCND2 in cumulus cells and its effect on oocyte maturation. ATP6 is part of the mitochondrial ATP synthase complex, which plays a key role in ATP production. Although its exact function in cumulus cells remains unclear, recent studies suggest its potential involvement in energy metabolism. Overexpression of ATP6 in cumulus cells was associated with improved oocyte developmental potential. Higher levels of ATP6 in COCs are associated with increased oocyte quality and their capacity for fertilization and subsequent embryonic development [60]. Our results align with these findings. We observed increased expression of these specific genes in oocytes matured in FLI medium, particularly SF FLI group. This highlights the impact of cumulus cells and maturation medium on oocyte developmental competence.

## Conclusions

The results of this research indicate that the addition of FGF2, LIF, and IGF1 to the culture medium can enhance both the meiotic and developmental capabilities of pig oocytes at different developmental stages, including those that have not completed their meiotic maturation, but achieved full-size. Additionally, it accelerates the transformation of oocyte chromatin from a diffuse pattern to a condensed one, leading to highly synchronized maturation rates for SF and LF oocytes. Implementing this, oocyte culture technique has the potential to significantly boost the quantity of oocytes suitable for in vitro maturation and subsequent assisted reproductive technologies (ART), specifically in pigs. Verifying the effectiveness of this methodology on other mammalian species would be an interesting area for further investigation.

### Electronic supplementary material

Below is the link to the electronic supplementary material.

Supplementary Material 1

Supplementary Material 2

## Data Availability

No datasets were generated or analysed during the current study.
