# Supplementary material for: Meiotic and developmental competence of growing pig oocytes derived from small antral follicles is enhanced in culture medium containing FGF2, LIF, and IGF1 (FLI medium)
Source: J Ovarian Res. 2024 Mar 2;17:54. doi: 10.1186/s13048-024-01360-0 (PMC10908066; doi:10.1186/s13048-024-01360-0)
Supplement: Supplementary file 1 — Supplementary Material 1 [file 13048_2024_1360_MOESM1_ESM.docx]

Supplemental Table 1. Maturation of pig oocytes derived from small and large follicles in control, FLI and FCS-supplemented medium.

(FLI promotes maturation of pig oocytes derived from small follicles)

| Type of  medium | Type of donor  follicle | No. of oocytes  examined | No. of oocytes in | | |
| --- | --- | --- | --- | --- | --- |
|  |  |  | GV (%) | GVBD (%) | MII (%) |
| Control | Small | 139 | 20.86±0.77 | 27.33±4.05 | 49.64±1.6^a^ |
|  | Large | 128 | 6.25±4.55 | 14.06±2.89 | 79.68±0.47^b^ |
| FLI | Small | 121 | 7.43±1.63 | 14.87±1.31 | 77.68±4.1^a^ |
|  | Large | 123 | 3.25±1.03 | 9.75±1.04 | 86.99±2.4^a^ |

Control: M199 supplemented with BSA, PMSG, hCG and EGF. FLI: Control medium supplemented with FGF2, LIF and IGF1. GV: germinal vesicle; GVBD: germinal vesicle breakdown; MII: metaphase II. Data are expressed in percentages ± SEM. Values with different superscript are significantly different within the column (P<0.01).
