# Supplementary material for: Meiotic and developmental competence of growing pig oocytes derived from small antral follicles is enhanced in culture medium containing FGF2, LIF, and IGF1 (FLI medium)
Source: J Ovarian Res. 2024 Mar 2;17:54. doi: 10.1186/s13048-024-01360-0 (PMC10908066; doi:10.1186/s13048-024-01360-0)
Supplement: Supplementary file 2 — Supplementary Material 2 [file 13048_2024_1360_MOESM2_ESM.docx]

Supplemental Table 2. Developmental potential of oocytes derived from small and large follicles in control and FLI medium

| Type of  medium | Type of donor  follicle | No. of oocytes  examined | Cleavage rate | Blastocyst rate |
| --- | --- | --- | --- | --- |
| Control | Small | 144 | 44.47±0.84 | 10.89±1.93^a^ |
|  | Large | 148 | 71.64±2.61 | 23.62±1.61^b^ |
| FLI | Small | 150 | 77.33±8.21 | 29.19±2.09^a^ |
|  | Large | 150 | 80±3.26 | 31.63±1.36^a^ |

Control: M199 supplemented with BSA, PMSG, hCG and EGF. FLI: Control medium supplemented with FGF2, LIF and IGF1. GV: germinal vesicle; GVBD: germinal vesicle breakdown; MII: metaphase II. Data are expressed in percentages ± SEM. Values with different superscript are significantly different within the column (P<0.01).
